# Prolonged recovery of 3D printed, photo-cured polylactide shape memory polymer networks

**DOI:** 10.1063/5.0008910

**Published:** 2020-08-20

**Authors:** Alberto Di Bartolo, Ferry P. W. Melchels

**Affiliations:** Institute of Biological Chemistry, Biophysics and Bioengineering, School of Engineering and Physical Sciences, Heriot-Watt University, EH14 4AS Edinburgh, United Kingdom

## Abstract

Shape memory polymers are materials that are able to retain a deformed state until an external stimulus, most typically heat, triggers recovery to the original geometry. Whereas typically, shape memory polymers are required to recover fast (seconds to minutes), many applications, particularly in the medical field, would benefit from a slow recovery (days to weeks). In this work, we exploit the broad glass transition range of photo-cured poly(D,L-lactide) dimethacrylate networks to obtain recovery times of up to 2 weeks, at 11 °C below the peak glass transition temperature of 58 °C. Recovery times decreased considerably for higher recovery temperatures, down to ∼10 min at 55 °C. A large spread in glass transition values (53.3–61.0 °C) was observed between samples, indicating poor reproducibility in sample preparation and, hence, in predicting shape recovery kinetics for individual samples. Furthermore, a staged recovery was observed with different parts of the samples recovering at different times. The ability to prepare complex structures using digital light processing stereolithography 3D printing from these polymers was confirmed. To the best of our knowledge, this work provides the first experimental evidence of prolonged recovery of shape memory polymers.

## INTRODUCTION

Shape memory polymers (SMPs) can be deformed and fixed into a temporary shape, to later be triggered into recovering to the original geometry by the application of a certain stimulus.^1,2^ The possible stimuli include heat,^1^ light irradiation,^3–7^ electric currents,^8–10^ magnetic fields,^11,12^ solvents,^13–17^ pH change,^18,19^ and redox reactions.^20,21^ Most SMPs are triggered by direct heating and, hence, referred to as thermally actuated or thermoresponsive. Examples of such polymers are SMPs that typically hold their temporary shape upon cooling through crystallization below their melting temperature [e.g., poly(cyclooctene)^22^] or vitrification below their glass transition temperature [e.g., sodium montmorillonite poly(ethyl methacrylate) (PEMA) nanocomposites^23^]. The permanent shape is dictated by (mostly covalent) crosslinks, and hence, this usually is the shape in which the polymer network was prepared. The crosslinks act as netpoints that are not affected by the change in temperature applied to the polymer when triggering the shape recovery. They provide the overall geometrical stability required for the original, permanent, shape to be recovered. The shape fixation and subsequent recovery of vitrification-based SMPs rely on a difference in segmental mobility of several orders of magnitude at either side of the glass transition region. The deformation can be fixed by vitrification at sufficiently low temperature (typically at 30 °C or more below T_g_), and the original shape can be recovered once the polymer is heated up to its T_g_ because of increased mobility and entropy elasticity.^1^ A relatively low density of covalent crosslinks is sufficient for retention of the permanent shape, while having a limited influence on segmental mobility. This allows for the shape memory behavior to occur.

SMPs present appealing properties that can be exploited to obtain smart medical devices. Temporary compacted devices can be deployable by minimally invasive surgery and can potentially be resorbed in the body after fulfilling their function. Early research by Lendlein and Langer focused on investigating biocompatible SMPs based on polycaprolactone (PCL),^24,25^ which later included degradability, and their potential use in self-tightening sutures.^26^ Other biomedical applications reported include self-deployable embolization devices,^27,28^ stents,^29,30^ drug-eluting devices,^31^ and self-expanding scaffolds for tissue engineering.^32,33^ The research on biomedical SMPs also includes studies on cellular solid design of SMP foams^34^ and on the influence of sterilization on shape memory behavior.^28,35^ Most of the reported applications require fast recovery, and in many studies, the time taken for shape recovery has not been a main focus of the research. However, chemically crosslinked amorphous networks acting as SMPs lend themselves well for fitting models of viscoelasticity, thereby allowing full thermomechanical characterization.^36–43^ Theoretical and experimental research assessed the influence of several parameters on shape recovery kinetics. A virtual “reduced programming time” was identified based on the time-temperature superposition principle, which encapsulates the holding time and temperature used in the programming phase and can be used to predict shape fixity and free recovery behavior.^41^ Shape recovery times of up to 2 h were reported in this and similar studies.^39,40,44^

In this work, we aim to utilize the inherently broad glass transition of chain crosslinked polymer networks to achieve a much longer recovery behavior. We hypothesize that by controlling the glass transition of crosslinked amorphous polymer networks with respect to the recovery temperature, recovery lasting up to days or weeks may be achieved.

We envisage many novel and valuable applications of SMPs with prolonged recovery times in the biomedical field. These include slow-deploying stents for gradual dilation of blood vessels or urethras, allowing the surrounding tissue to adjust and grow as the stent expands. The dilation of urethra strictures by a single slow-deployable stent may be much more cost-effective and less invasive than the current treatment, where an inflatable balloon or thin rods of increasing diameters are inserted into the urethra in order to open the urethral narrowing.^45^ Similarly, the repeated dilation of blood vessels using balloon expandable stents is current clinical practice in growing pediatric patients and to counteract the effects of intimal hyperplasia in adults.^46^ Another similar application would be as tissue expanders. Currently, silicone shells are implanted under the skin for up to several months and filled with regular injections of saline to expand the skin while allowing it to grow, for example, prior to breast reconstructions.^47^ Such procedures may be replaced by a single implantation of a slowly deployable expander; porous shape memory foams can be compacted as much as 80% and still experience full strain recovery.^48^ A similar application would be the expansion of skin prior to ear reconstruction in microtia patients.^49^ Even more advanced applications can be envisioned, for example, in scaffolds for tissue engineering that would gradually increase in size and volume over the course of weeks, allowing cells to proliferate and fill the scaffold or surrounding tissue (including vasculature) to grow inward steadily. One study looked at the effect of shape recovery on cells seeded on a SMP scaffold and concluded that a single mechanical stimulus over 30 min was sufficient to initiate changes in the morphology of adherent cells.^50^ This sparks the question if tissue growth and organization can be further influenced by longer term recovery, fitting within the theoretical framework of developmental engineering.^51^ Furthermore, the ability to 3D print SMPs with prolonged recovery would create additional opportunities for medical use because tissue expanders and other medical devices could be made patient-specific. Stereolithography or digital light processing (DLP) 3D printing has been previously used to produce chemically crosslinked polymers with inherent shape memory behavior.^52^

Here, we studied the recovery kinetics of crosslinked poly(D,L-lactide)dimethacrylate networks at different temperatures below the peak glass transition temperature, over several weeks. The observed behaviors were related to thermomechanical properties obtained from temperature sweeps using dynamic thermomechanical analysis (DMTA). Furthermore, the ability to prepare such networks into complex shapes using stereolithography 3D printing on a low-cost DLP printer was confirmed.

## RESULTS

### Preparation and dynamic mechanical analysis of polylactide SMP networks

The synthesis of poly(D,L-lactide)dimethacrylate (PDLLA-2MA) macromonomers with a molecular weight M_n_ of 2.85 ± 0.18 kg/mol, a D,L-lactide (DLLA) monomer conversion of 97% ± 2.2%, and a degree of methacrylation of 94% ± 1.8% was confirmed through ^1^H-NMR (supplementary material Fig. 2). These macromonomers were used in the formulation of a liquid resin, further containing a non-reactive diluent/solvent (benzyl alcohol) and a photo-initiator (TPO), from which rectangular strips were photo-polymerized on a low-cost DLP stereolithography 3D printer. These strips were used in all thermomechanical and shape recovery studies reported, while the same resin, was used to print porous 3D structures as described later.

**FIG. 1.**
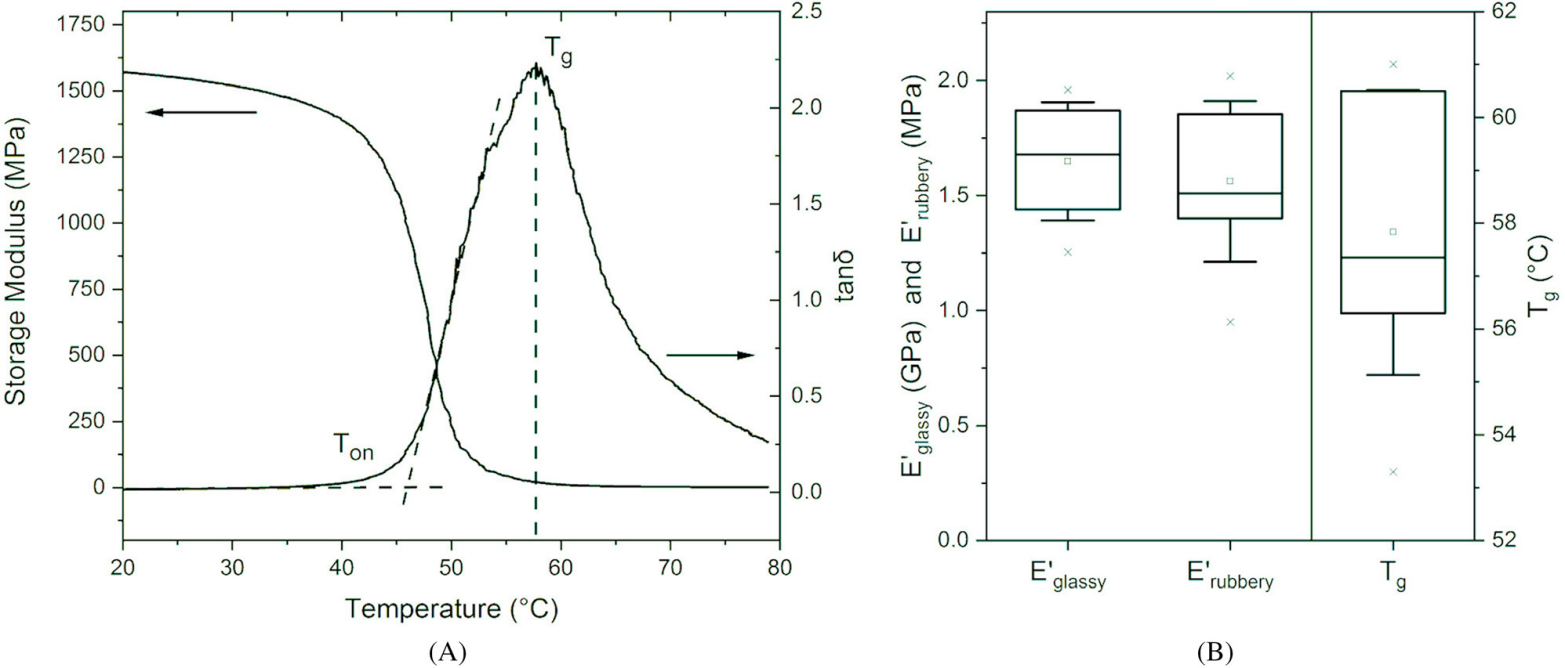
(a) Storage modulus and tan δ curves for the shape memory networks, as obtained from dynamic mechanical analysis using a temperature sweep. (b) Box plots for thermomechanical data of eight specimens of PDLLA-2MA networks prepared from the same resin; glassy storage modulus, rubbery storage modulus, and peak glass transition temperatures. For each plot, the box range is 25th–75th percentile, the horizontal line is the median, the thin-lined □ symbol marks the mean, × symbols mark the full range, and whiskers mark the mean ± standard deviation.

Figure 1(a) reports the curves of storage modulus (E′) and damping factor (tan δ) against temperature for a representative PDLLA-2MA network, as obtained from a temperature sweep test on a dynamic mechanical analyzer in tension mode. The observed storage modulus curve is typical for chemically crosslinked polymers.^54,55^ The plateau at lower temperatures is the glassy plateau, where the storage modulus is on the order of GPa (e.g., 1.57 GPa at 20 °C). The plateau at higher temperatures is the rubbery plateau, with a storage modulus of about three orders of magnitude lower (e.g., 1.57 MPa at 79 °C). The tan δ curve is broad (width at a half maximum intensity of 14 °C) as typically observed for chain crosslinked networks,^56^ forming the basis of our hypothesis that slow and predictable recovery may be obtained. The peak of the curve corresponds to a temperature of 58 °C, which is taken as the glass transition temperature T_g_. The onset of the curve is taken as marking the beginning of the glass transition region. It is measured as the intersection of the dashed straight lines and here corresponds to a temperature T_on_ of 46 °C.

Figure 1(b) shows the distribution of glassy storage modulus, rubbery storage modulus, and peak glass transition temperatures obtained from eight independent temperature sweeps, on eight samples prepared from the same resin. It is noted that the spread of values is large, particularly for Tg (53.3–61.0 °C). Differential Scanning Calorimetry (DSC) was used to confirm that the large scatter was indeed caused by sample-to-sample variation, rather than being an artifact from the Dynamic Mechanical Analysis (DMA) measurements (supplementary material Fig. 3). The importance of the large spread in T_g_ values for shape memory recovery kinetics will be elucidated later on.

### Shape memory programming and recovery near the glass transition temperature

**FIG. 2.**
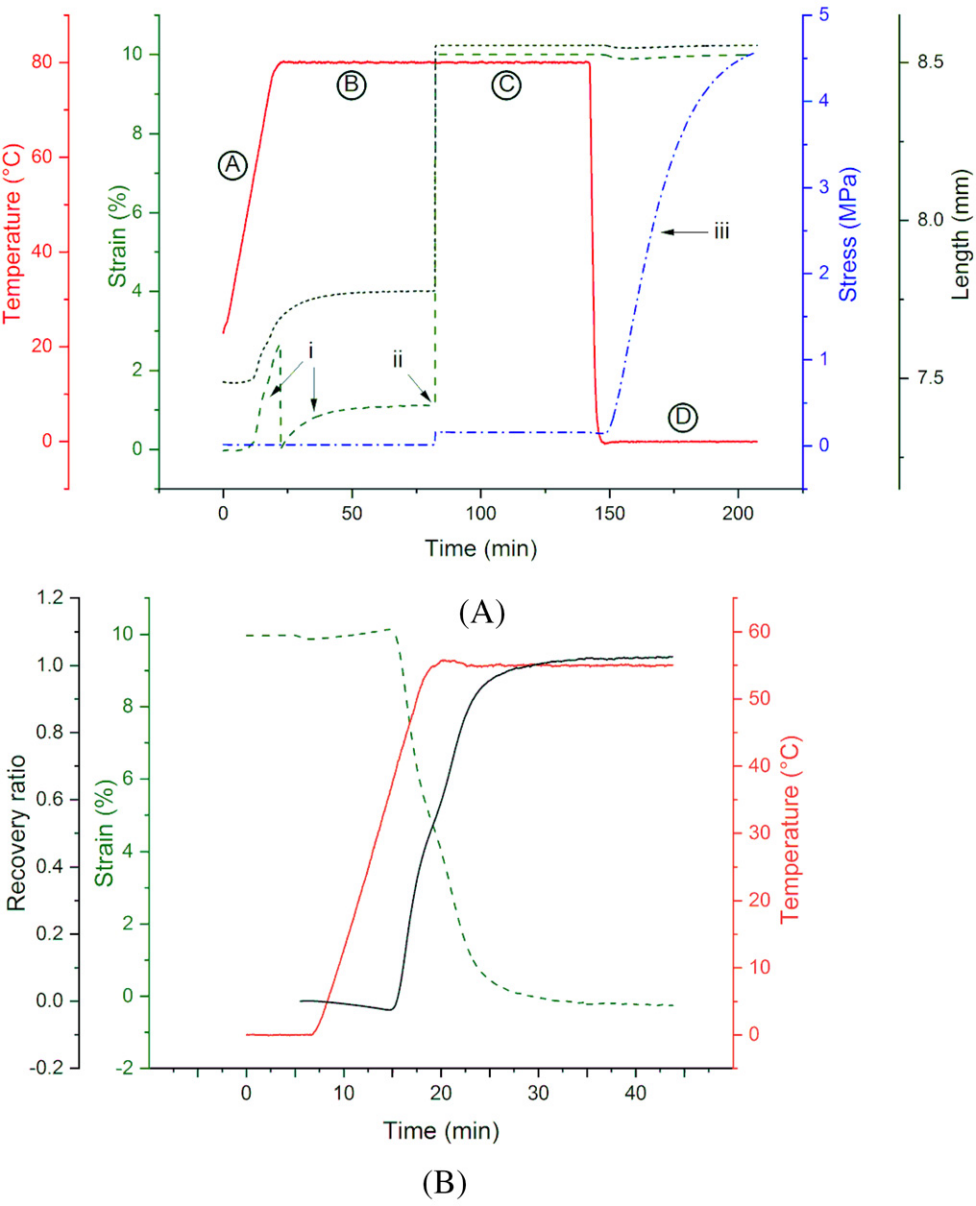
(a) Example of shape programming. The capitalized letters denote the different steps during programming: (A) temperature ramp from room temperature to T_h_ (80 °C); (B) 60 min isothermal step to complete equilibration; (C) 60 min isothermal and isostrain step following loading to achieve 10% strain; (D) cooling ramp to Tc (0 °C) followed by 60 min isothermal. Roman numerals represent points of interest: (i) strain increase during the equilibration step; (ii) instantaneous loading; (iii) stress increase during the cold temperature isotherm. (b) Strain, temperature, and recovery ratio evolution for free recovery at 55 °C of a PDLLA-2MA shape memory network programmed with a 10% tensile strain as shown in (a).

Based on the broad glass transition region observed above, we chose to study shape recovery of our photo-polymerized polylactide SMP networks at several temperatures between T_on_ and T_g_. For a recovery temperature (T_rec_) of 55 °C, the specimen was programmed entirely on the DMA, following the shape memory programming cycle shown in Fig. 2(a). The cycle consists of a heating step (A), an equilibration step at 80 °C to remove thermal history (B), followed by isothermal (80 °C) and isostrain (10%) programming (C) and cooling (to 0 °C) to fix the temporary shape (D). An initial strain increase can be observed during heating and equilibration (i), which is accounted for by resetting the strain just before applying the deformation (ii). The observed strain increase can be ascribed to both heat expansion and the effect of the thermo-mechanical history during sample preparation. The duration of the equilibration step is such that the thermal history is completely removed, with the strain plateauing in around 30 min. During the cooling step (D), a considerable increase in stress can be observed (iii). This phenomenon is possibly related to thermal contraction, as the strain is kept constant at 10%.

Figure 2(b) reports the temperature, strain, and recovery ratio curves during the free recovery step of a network programmed as described above. Here, free recovery was followed on a DMA at a recovery temperature (T_rec_) of 55 °C, which is close to the observed T_g_. As typical for any SMP, a small amount of strain (Δε) is lost right after unloading; this drop in strain determines the shape fixity ratio (R_f_) as per the following equation:

$$R_{f}=\frac{\varepsilon_{\text{max}}-\Delta \varepsilon }{\varepsilon_{\text{max}}}.$$ (1)

For our network, programmed as explained above, 0.3% out of 10% maximum applied strain is lost after unloading; hence, the shape fixity is 97%. Upon heating, the strain initially increases and then starts to decrease once the temperature in the chamber reaches 38 °C. The deformation is then recovered in 10 min, with a final asymptotic strain slightly lower than 0%, with a value of −0.2%. The values of recovery ratio were calculated from the strain evolution, *ε*(*t*), and from the value of strain right after unloading as per the following equation:

$$R_{r}(t)=1-\frac{\varepsilon (t)}{\varepsilon_{\text{max}}-\Delta \varepsilon }.$$ (2)

Similarly, though inversely proportional to the strain evolution, the recovery ratio initially decreases to values below 0 and then increases toward 1 as the temperature stabilizes. The asymptotic value of the recovery ratio is shown to be 1.02, meaning that out of the initially applied strain of 10%, 10.2% was recovered. In other words, the original sample length (100%), which was stretched to 110% during programming, first recovered to 109.7% upon unloading (97% shape fixity) and then further recovered to 99.8% of its original length upon heating.

### Prolonged shape recovery at lower temperatures

To test our hypothesis that PDLLA-2MA networks will show prolonged recovery at temperatures below T_g_ and nearer T_on_, we performed shape recovery experiments at 45, 48, 50, and 53 °C. For practical reasons, these long-term experiments were performed inside a temperature-controlled oven rather than the DMA. The specimens were programmed manually by the use of a clamping system, inside the same temperature-controlled oven [mimicking stages A, B, and C of the programming cycle shown in Fig. 2(a)] and a freezer (mimicking stage D). The maximum applied strain was calculated through image analysis of each specimen and equaled 9.18% ± 1.44% (*n = *6).

**FIG. 3.**
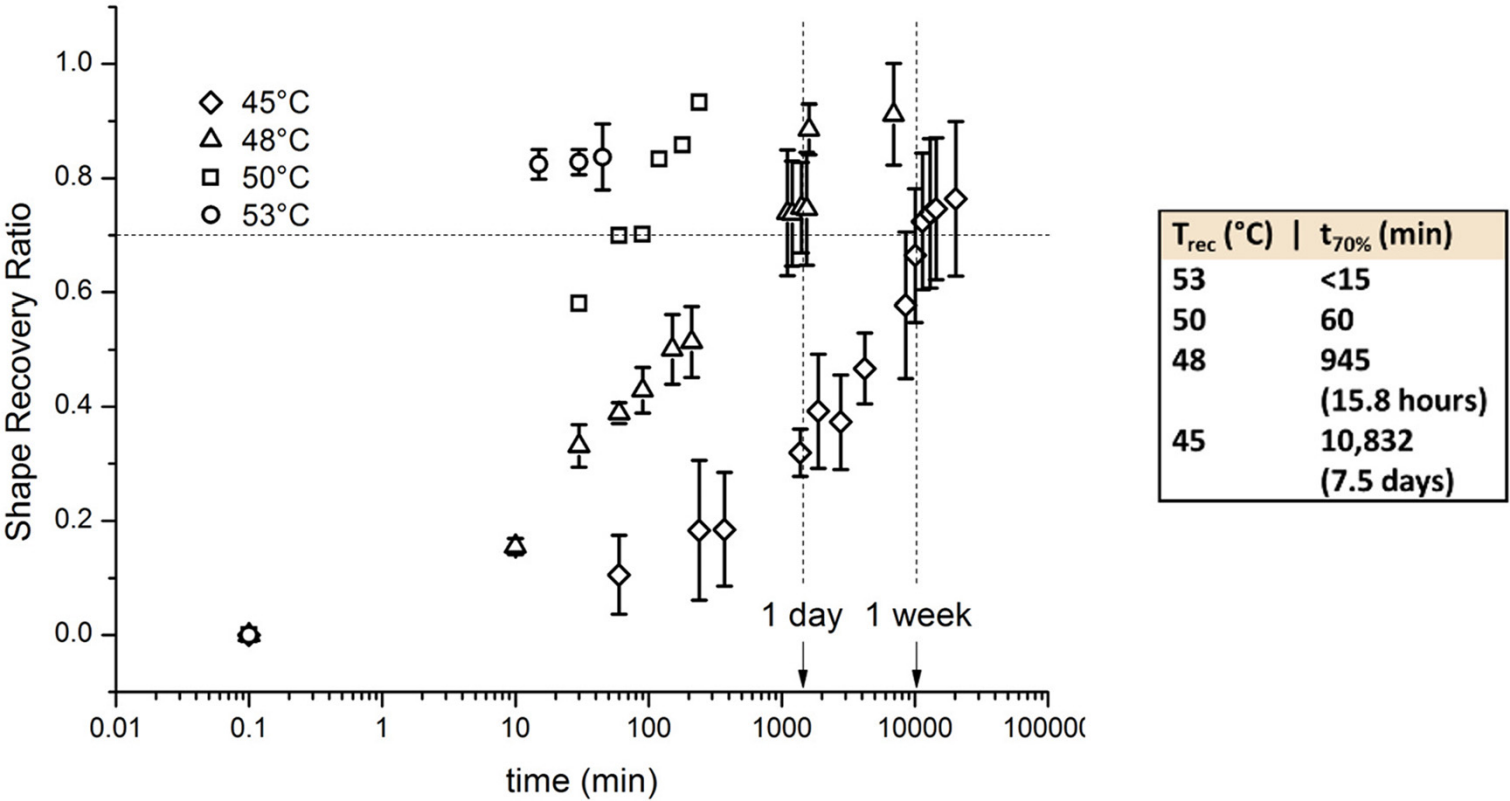
Shape recovery ratio evolution for PDLLA-2MA networks recovering isothermally at temperatures ranging from 45 to 55 °C. The shape recovery ratio was calculated based on an initial length measured just before elevating the temperature to the recovery temperature; the time at this point is arbitrarily plotted as 0.1 min. Note the logarithmic scale of the time axis. Data points and errors bars represent mean ± standard deviation; n = 3 for all temperatures except 50 °C, for which n = 1. The table insert shows the time taken to achieve 70% recovery (R_r_ = 0.7) for each temperature, corresponding to the horizontal dashed line in the graph.

Figure 3 shows representative shape recovery kinetics of PDLLA-2MA networks recovering isothermally at temperatures varying from 45 °C to 53 °C. The 55 °C curve in Fig. 2 is continuous as it was measured in continuous mode on the DMA, whereas at the other temperatures, the manual measurements gave rise to discrete points (Fig. 3). The values of strain were subsequently converted to discrete values of the recovery ratio by the equation

$$R_{r}(t_{j})=1-\frac{\varepsilon (t_{j})}{\varepsilon (t_{0})},$$ (3)

where *ε*(*t_j_*) is the discrete value of strain at a certain observation time (*t_j_*) and *ε*(*t*_0_) is the value of strain observed just before the specimen was moved to the oven. All evolutions show a similar overall profile. The rate of recovery starkly decreases with decreasing temperature. The table insert in Fig. 3 shows the time taken to achieve 70% shape recovery, at each of the investigated temperatures. This shows an increase in recovery time from less than 15 min at 53 °C, up to 7.5 days at 45 °C. Besides these temperatures, programmed samples were also left at 37 °C and room temperature (approximately 20 °C) for a minimum of 6 months, during which no recovery was observed, meaning that the sample length remained totally unchanged.

**FIG. 4.**
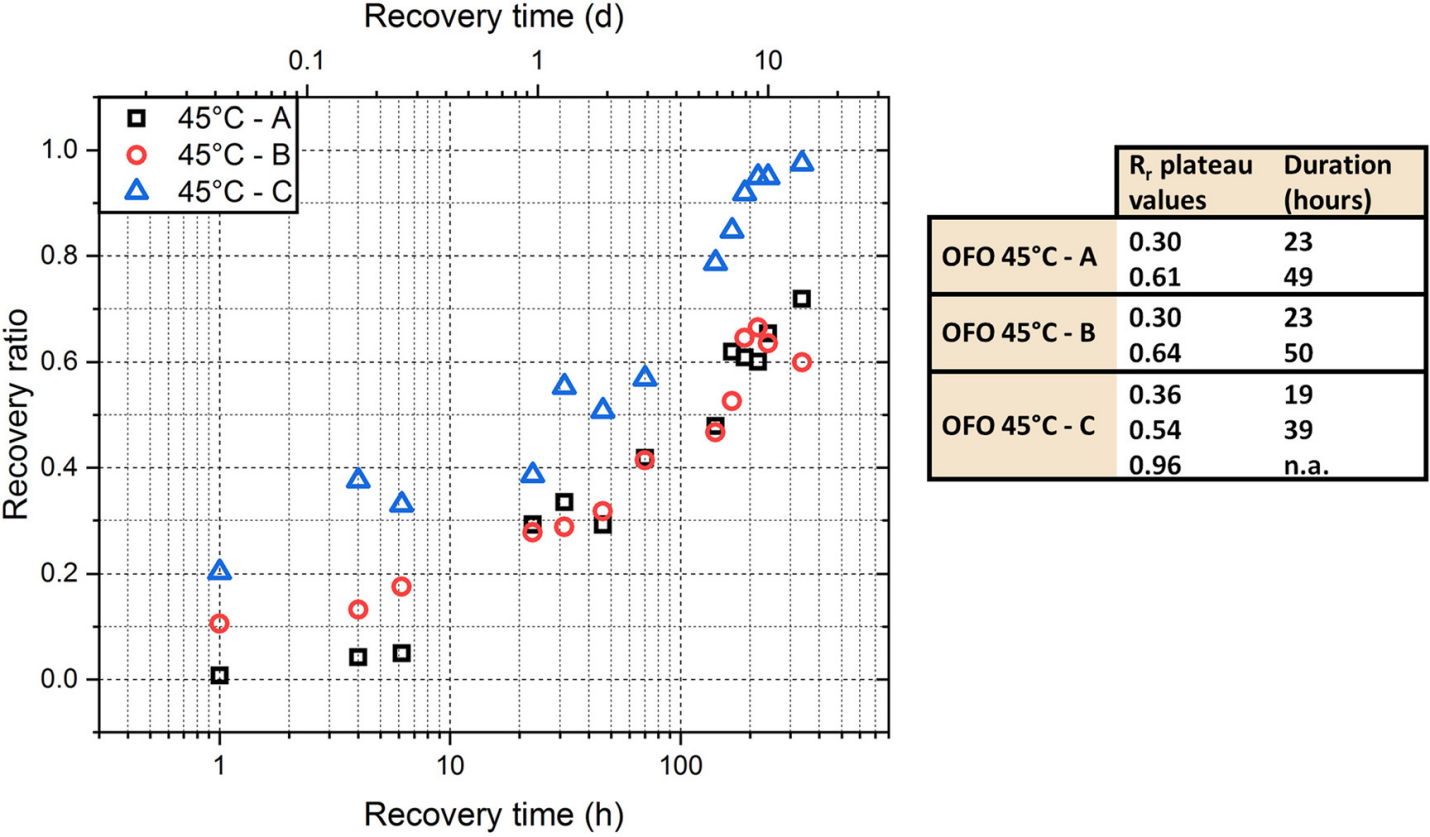
Shape recovery ratio evolution for three individual PDLLA-2MA network samples recovering isothermally at 45 °C over 14 days (i.e., 336 h). The shape recovery ratio was calculated based on an initial length measured just before elevating the temperature to the recovery temperature; the time at this point is arbitrarily chosen as 0.1 min. Note the logarithmic scale of the time axis. The table insert shows the values of the recovery ratio at which a plateau was observed, as well as the time the plateau was observed.

Figure 4 shows the individual profiles for recovery ratios measured for three samples at a recovery temperature of 45 °C. It demonstrates that the error bars in Fig. 3 are a result of an offset between the individual profiles, rather than random scatter in the recovery ratio at each point. When comparing the individual recovery profiles, we notice how specimens A and B show very similar behavior from 24 h, though they do not share the same recovery values for the first 6 h of testing. During this early stage, specimens A and B recover 5% and 18% of deformation, respectively. Both specimens follow a similar behavior for all other data points and show a final value of recovery ratio lower than one. Specimen C shows a faster recovery behavior, it recovers more than 20% deformation over the first hour, and its final value of R_r_ is close to one.

For the three specimens, it was noticed that the recovery proceeds through a series of plateaus, during which the value of R_r_ is relatively constant. The table insert in Fig. 4 reports the average value of R_r_ and the duration of these plateaus. It is noticed that the values of R_r_ at which a plateau is encountered are similar for the three specimens, and the same can be inferred for the duration of the plateaus. For specimen C, a third plateau around a value of R_r_ equal to 0.96 is also reported, which is assumed to be the final plateau corresponding to the full recovery of the deformation.

### DLP stereolithography 3D printing

**FIG. 5.**
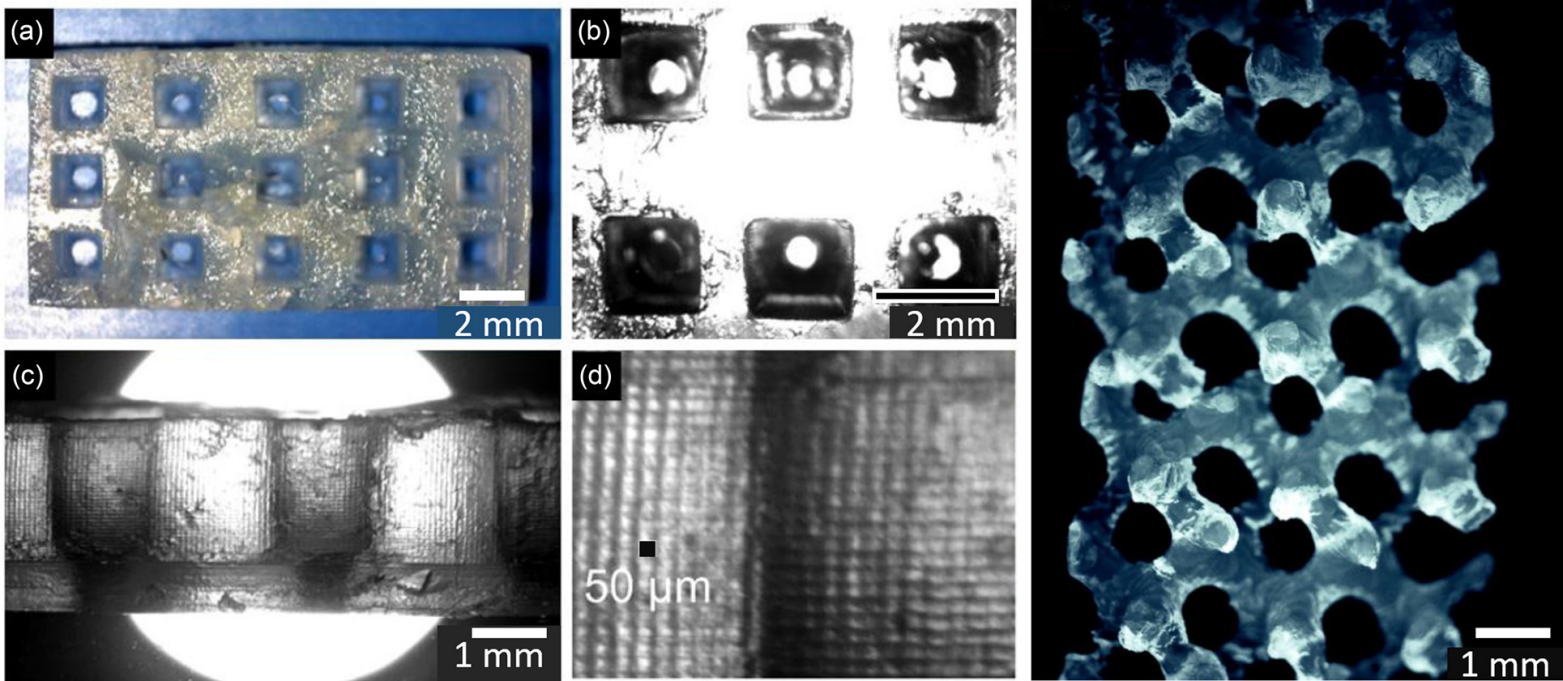
Results from printing test parts: (a) top view of the printed slab with a cross section of 15 × 7.5 mm^2^ and 1.5 × 1.5 mm^2^ rectangular straight pores; (b) top plane view under the microscope; (c) side view under the microscope showing the decreased pore size in the overexposed bottom layers; (d) magnification of picture (c) showing the voxel pattern; the black square represents one 50 × 50 *μ*m^2^ pixel; (e) top view of the gyroid geometry scaffold print test. The part covers an area of 5 × 10 mm^2^ containing 3 × 6 gyroid unit cells with a period of 1.6 mm.

The printability of this photo-curable polylactide SMP formulation was confirmed using a commercially procured, low-cost open source DLP printer (Autodesk Ember). The same resin in use for the preparation of the shape recovery samples was employed for the printing of simple structures, which is required for accurate layer-by-layer fabrication.^53^ Figure 5 shows results from printing test parts: (a) top view of the printed slab with a cross section of 15 × 7.5 mm^2^ and 1.5 × 1.5 mm^2^ rectangular straight pores; (b) top plane view under a microscope; (c) side view under a microscope showing the decreased pore size in the overexposed bottom layers; (d) magnification of picture c showing the voxel pattern; the black square represents one 50 × 50 *μ*m pixel; (e) top view of the gyroid geometry scaffold print test. The part covers an area of 5 × 10 mm^2^ containing 3 × 6 gyroid unit cells with a period of 1.6 mm, which shows two different parts fabricated, a rectangular slab with square through-pores (a)–(d) and a 70% porous gyroid structure (e). It can be noticed that in the slab, the pore edges are straight and the rectangular geometry is well reproduced (a)–(c). From the side view in image (c), it can be observed that the pores are partially occluded. This area corresponds to the first 10 layers that were printed at 20 s of light exposure to ensure good adhesion to the build-head and good mechanical strength of the part base. After these ten layers, the printing continued with 10 s of light exposure, which led to the correct reproduction of the designed pores. The layered structure can be observed, and from the magnification in panel (d), the voxel pattern forming the part can be noticed.

Shape memory behavior was observed qualitatively by manual compression of the porous gyroid scaffold after heating, followed by fixation upon cooling in cold water and recovery triggered by submersion in hot water (approximately 60 °C). Quantitative characterization of the shape memory kinetics will be the subject of future studies.

## DISCUSSION

With this work, we demonstrated that the broad glass transition of amorphous polymer networks can be utilized to obtain prolonged shape recovery. At temperatures close to the peak glass transition temperature, the observed recovery lasted several minutes. Conversely, the recovery time increased up to 2 weeks when performing the recovery at lower temperatures, nearer the onset temperature of the glass transition area. Below this onset temperature (i.e., at 37 °C and 20 °C), no noticeable shape recovery was observed over months.

To the best of our knowledge, this is the first demonstration of such long recovery. Most research on SMPs is focused on the shape fixity and ultimate recovery ratio rather than on the kinetics of recovery. This is perhaps because of many applications requiring fast or even instant recovery.^1,4,10,29,57^ Work that has been done on modeling, predicting, and demonstrating kinetics of shape recovery has typically covered much shorter recovery times.^36,39,41,43^ Yu *et al.* elegantly demonstrated how the holding time and temperature during programming can be used to influence the free recovery kinetics in a mathematically defined manner, but the same mathematical model can also be used to demonstrate that this method of tailoring recovery kinetics is limited to recovery times of up to a few hours only.^41^ The longest studied recovery time we have come across in the literature was 12 h, on shape memory polyurethane foams.^28^

As the recovery temperature for biomedical applications is set at 37 °C, the glass transition range will have to be adjusted to obtain the desired recovery rate under physiological conditions. Besides the materials reported here, SMP networks were also prepared in which a part of the lactide was replaced by ε-caprolactone (the polymer of which has a T_g_ value of −60 °C), resulting in networks with peak T_g_ values in the range of 30–47 °C and similar broad glass transitions (supplementary material Fig. 4). However, for reasons of accuracy and convenience, for this study, we opted to characterize one polymer (PDLLA-2MA network) in a range of recovery temperatures, rather than vice versa.

A major obstacle in the application of these SMPs for prolonged recovery is the large variability in thermomechanical properties between samples. Even when using a single batch of resin made from a single batch of macromonomers, samples prepared from it exhibited T_g_ values ranging from 53.3 to 61.0 °C. As a difference in the recovery temperature of only 3 °C (45 vs 48 °C) already caused a full order of magnitude difference in recovery time, this poor reproducibility of T_g_ is prohibitive for applications where predictable recovery times are required. One possible cause for such spread in thermomechanical properties is the inherently heterogeneous nature of chain crosslinked polymer networks. Chain cross-linking of difunctionalized macromonomers tends to proceed through the formation of microgels in the early stage of polymerization, which are connected into a single network at a later stage.^58^ In combination with a broad distribution of polymethacrylate kinetic chain lengths, this leads to heterogeneously crosslinked networks.^59^ A second possible cause is related to the photo-initiation of the cross-linking reaction. In photo-initiated polymerization, the light intensity decreases with the depth into the reaction mixture, causing gradients in initiation rates, and hence, a spatial variation in conversion, the kinetic chain length, and cross-link density can be expected.^60^ The gradient in conversion along the specimen depth would also explain why some specimens go through a bent shape in the course of recovery (supplementary material Fig. 1), as there would be a difference in the recovery rate at the two faces of the specimen because of different cross-linking degrees. In addition to the gradient along the specimen depth, the light intensity across the printer window is not perfectly uniform, which would also result in gradients in conversion and cross-link density in other directions. The network inhomogeneity may also have caused the start-stop behavior in recovery. This behavior was evidenced by plateaus in the recovery profile, where all three samples spent similar periods of time with little recovery (19–23 h for the first plateau and 39–50 h at the second) in-between periods of greater recovery. These occurred at similar stages during the recovery, i.e., at 30%–36% recovery for the first plateau and at 54%–64% for the second plateau. This behavior is similar to multiple-shape memory polymers, where different recovery kinetics are activated as time goes by, reflecting areas of varying segmental mobilities due to small differences in the network topology.^61–63^

Better defined, more homogeneous polymer networks could be obtained by changing both the cross-linking chemistry (e.g., a click reaction or other step-growth mechanism)^64^ and the mode of initiation (e.g., chemical or thermal). It is highly recommended to perform thermomechanical studies as performed here on such SMPs, to better understand the true potential of slow-recovering SMPs. Changing the chemistry and, more so, initiation mode will preclude direct DLP 3D printing as a technique to prepare designed structures from predictably slow-recovering SMPs, but other 3D printing techniques and indirect stereolithography 3D printing (via a 3D printed mold) could be explored.

The ability for our PDLLA-based SMPs to be 3D printed into designed shapes using DLP stereolithography was confirmed using a low-cost commercial printer. The ability to employ these SMPs in 3D printing enables complex shapes including patient-specific implants to be prepared from slow-recovering SMPs. 3D printing was not the main focus of the study; therefore, it was not optimized nor characterized in detail. Several challenges were met, most notably the inability of the employed printer to reliably separate the build tray from the latest cured layer, which resulted in jamming. However, the ability to 3D print these macromonomers with non-reactive diluents was demonstrated previously^53,65^ on a more professional DLP printer. Those and other studies also demonstrated the lack of toxicity of these polymers and their application as scaffolds for 3D cell culture.^66,67^ Here, we studied these highly similar materials in a new setting as shape memory polymers, demonstrating their prolonged recovery over more than one week.

## CONCLUSIONS

In this study, we demonstrated, for the first time, prolonged recovery of shape memory polymers up to 2 weeks. The recovery rate was found to depend strongly on temperature, with recovery ranging from minutes near the peak T_g_, (∼58 °C) to weeks near the onset of the glass transition range (∼46 °C). Below this onset temperature, no recovery was observed over months (37 °C and 20 °C), hence confirming the fixed state of the shape memory polymer. The chemistry and preparation method employed resulted in a large scatter in thermomechanical properties, excluding the possibility to predict or tailor the shape recovery kinetics accurately. Furthermore, the recovery was observed to proceed in a stop-start manner, similar to multiple-shape memory polymers. Finally, the ability to 3D print these polymers into complex shapes by DLP stereolithography was demonstrated. If more homogeneous polymer networks can be prepared in a more reproducible manner in the future, such shape memory polymer networks with prolonged recovery may open up a range of novel applications in the medical field.

## METHODS

### Materials

D,L-lactide (DLLA), PURASORB^®^ DL, was obtained from Corbion (The Netherlands); 1,6-hexanediol, tin(II)2-ethylhexanoate (SnOct_2_), methacrylic anhydride (MAAh), diphenyl(2,4,6-trimethylbenzoyl)phosphine oxide (TPO), 2,5-bis(5-tert-butyl-benzoxazol-2-yl)thiophene (BBOT), and hydroquinone (HQ) were acquired from Sigma Aldrich (UK); potassium carbonate (K_2_CO_3_), tetrahydrofuran (THF), and 2-propanol (IPA) were obtained from Fisher Scientific (UK). Benzyl alcohol (BnOH) was obtained from Alfa Aesar (UK).

### Macromonomer synthesis

Poly(D,L-lactide)dimethacrylate (PDLLA-2MA) macromonomers were synthesized similar to previously reported protocols.^53^ In short, PDLLA diol oligomers were synthesized through the ring opening polymerization of DLLA using hexanediol as an initiator and SnOct_2_ as a catalyst, at 130 °C for 72 h under a nitrogen atmosphere. For all batches, the same molecular weight of 3000 g/mol was targeted through the ratio of DLLA to hexanediol. Polymerization was followed by methacrylation of the hydroxyl groups at room temperature for 5 days in dry THF solvent, using an excess of 50–100 mol. % of MAAh per hydroxyl group and the same molar amount of K_2_CO_3_ as a proton scavenger. The macromonomers were purified by precipitation in cold (−80 °C) IPA, followed by water washing (24 h at 4 °C), filtration, and freeze-drying. Macromonomers were vacuum-sealed and stored at −20 °C until further use. The conversion of DLLA, degree of polymerization, and degree of methacrylation were confirmed by proton-nuclear magnetic resonance spectroscopy (^1^H NMR, CDCl_3_, Bruker AVIII 300 MHz).

Poly(D,L-lactide-*ran*-ε-caprolactone)dimethacrylate macromonomers of the same target molecular weight were prepared in the same manner, with the only difference being that a part of the DLLA monomer was substituted by the ε-caprolactone monomer in the ring opening polymerization.

### Sample preparation

PDLLA-2MA macromonomer (at 58.6 wt. %), TPO photo-initiator (at 2 wt. %), BBOT UV-absorber (at 0.2 wt. %), and HQ inhibitor (at <0.1 wt. %) were stirred in BnOH under gentle warming until completely dissolved to prepare photo-curable liquid resins. Glass microscopic slides of 73 × 52 mm were covered in a uniform thickness of 500 *μ*m of resin with a casting knife (Elcometer 3580). The slide was placed on the window of an Ember DLP printer (Autodesk, 405 nm, 22.5 mW/cm^2^) and cured for 60 s into films of 50 × 4 mm each. After curing, excess resin was absorbed on paper and rinsed away with IPA. The washed films were sandwiched between two sheets of Teflon (Kudo3D Inc., Titan Replacement Teflon Films), then between two glass slides, followed by post-curing for 20 min from each side in a UV-box (UVP crosslinker CL-1000L, 356 nm, 3 mW/cm^2^). Subsequently, the films were extracted in IPA in a Soxhlet apparatus for at least 72 h, left to deswell and dry gradually in saturated IPA vapor with excess liquid IPA, and finally dried at 80 °C in an oven (SciQuip SQ-4845) on Teflon sheets (RS Components Ltd.) for at least 72 h or until dry (i.e., negligible mass changeover 24 h). After extraction and drying, specimens measured 40 × 3.4 × 0.25 mm.

### Thermal characterization and shape memory programming

All thermo-mechanical characterization studies were performed on a DMA Q800, dynamic thermal mechanical analyzer (TA Instruments) equipped with an ACS-3 air chiller for sub-ambient temperature control.

Temperature sweep tests were performed in tension mode (films, clamped length 7.5 mm), in the multi-frequency—strain module with a preload of 0.01 N and amplitudes of 1 Hz and 15 *μ*m. The method consisted of 15 min equilibration steps at either 80 °C or 0 °C, and ramps up/down at 2°/min in-between, for three cycles. Only the data from the last cooling and heating ramps are reported.

Differential scanning calorimetry was performed on a DSC 2010 (TA Instruments) with aluminum pans and lids (TA Instruments, DP-TA-STD) on samples of 10–15 mg. Two runs from room temperature to 80 °C at 5 °C/min were performed, by cooling the chamber at an uncontrolled rate using ice and equilibration at 25 °C in-between. The data from the second heating ramp were analyzed.

### Shape recovery characterization

The shape memory programming and shape recovery characterization were performed on the same DMA Q800 or in a laboratory oven. Shape memory cycles on the DMA were performed with the same initial settings as above, using the following method: ramp to 80 °C at 3 °C/min; equilibrate at 80 °C and then isothermal for 60 min; impose a strain of 10%; isothermal for 60 min; equilibrate at 0 °C and then isothermal for 60 min; set force to 0 N; isothermal for 1 min; ramp to recovery temperature at 5 °C/min; isothermal for the duration of the experiment. The sample dimensions for DMA characterization were 10 × 3.4 × 0.25 mm^3^.

Oven/freezer/oven (OFO) shape memory cycles were performed manually with the use of a laboratory oven (SciQuip, SQ-4845) and a laboratory freezer (−20 °C) for long-term experiments at lower recovery temperatures. Film specimens (40 × 3.4 × 0.25 mm^3^) were labeled and marked for dimensional reference as in supplementary material Fig. 1, and images (about 2200 × 500 pixels) were taken at relevant time points to be processed using ImageJ (https://imagej.nih.gov/ij/). The lengths were also measured using a caliper (Mitutoyo 150 mm Digital Caliper). The films were equilibrated in the oven for one hour at 80 °C and then left to stretch under a 0.08 N force by clamping both ends with binder clips (still at 80 °C). Specimens were quenched in ice water while clamped, blotted dry, and kept in the −20 °C freezer for at least one hour. Recovery was performed in a pre-equilibrated oven, after unloading by removal of the clip. Measurements and images were taken: after equilibration at 80 °C, after one hour deformation (once quenched), before the start of the recovery process, and at different time points during the recovery process. While shape recovery experiments at 45, 48, 50, and 53 °C were performed in the oven, other specimens were stored at 37 °C (in an incubator) and 20 °C (in a temperature-controlled microscopy room). At the end of the experiments, all specimens were heated up (80–100 °C) for around 30 min and then left to cool down. Any residual strain recovery was measured using the caliper. For each recovery temperature, measurements were performed on samples that were prepared and post-processed together, from a single batch of resin.

### DLP stereolithography 3D printing

To improve printability, the previously described resin was modified by decreasing the concentration of PDLLA-2MA macromer to 48.9 wt. % to reduce viscosity and by decreasing the concentration of BBOT to 0.1 wt. % to allow for larger overcure in order to improve layer-to-layer adherence. The Autodesk Ember was employed as per the manufacturer's instructions, with one modification to reduce attachment between printed part and the window. A Teflon sheet (Kudo3D) was cut to size, laid on top of the window, and held firmly in place by surface tension without requiring active adhesion (similar to a mobile phone screen protecting sheet). The computer-aided design file for the rectangular slab was produced on AutoCAD (Autodesk), while a part of 3 × 6 × 12 gyroid unit cells was produced on k3dsurf (http://k3dsurf.sourceforge.net/).

### Ethics

No ethics approval was required for this work.

## SUPPLEMENTARY MATERIAL

See the supplementary material for the supporting figures referred to in this manuscript [photographs, NMR spectra, DSC data, and data on poly(D,L lactide-co-ε-caprolactone) copolymer networks].

## Data Availability

The data that support the findings of this study are available from the corresponding author upon reasonable request.
